# Genomic evaluation for breeding and genetic management in *Cordia africana*, a multipurpose tropical tree species

**DOI:** 10.1186/s12864-023-09907-z

**Published:** 2024-01-02

**Authors:** Kedra M. Ousmael, Eduardo P. Cappa, Jon K. Hansen, Prasad Hendre, Ole K. Hansen

**Affiliations:** 1 Department of Geosciences and Natural Resource Management, University of Copenhagen, Rolighedsvej 23, 1958 Frederiksberg C, Denmark; 2 Instituto Nacional de Tecnología Agropecuaria (INTA), Instituto de Recursos Biológicos, Centro de Investigación en Recursos Naturales, De Los Reseros y Dr. Nicolás Repetto s/n, 1686 Hurlingham, Buenos Aires, Argentina; 3 Consejo Nacional de Investigaciones Científicas y Técnicas (CONICET), Buenos Aires, Argentina; 4 World Agroforestry Centre (ICRAF), United Nations Avenue, Nairobi, 00100 Kenya

**Keywords:** Single-step GBLUP, Quantitative genetic parameters, *Cordia africana*, Tropical tree breeding, Genetic management

## Abstract

**Background:**

Planting tested forest reproductive material is crucial to ensure the increased resilience of intensively managed productive stands for timber and wood product markets under climate change scenarios. Single-step Genomic Best Linear Unbiased Prediction (ssGBLUP) analysis is a cost-effective option for using genomic tools to enhance the accuracy of predicted breeding values and genetic parameter estimation in forest tree species. Here, we tested the efficiency of ssGBLUP in a tropical multipurpose tree species, *Cordia africana,* by partial population genotyping. A total of 8070 trees from three breeding seedling orchards (BSOs) were phenotyped for height. We genotyped 6.1% of the phenotyped individuals with 4373 single nucleotide polymorphisms. The results of ssGBLUP were compared with pedigree-based best linear unbiased prediction (ABLUP) and genomic best linear unbiased prediction (GBLUP), based on genetic parameters, theoretical accuracy of breeding values, selection candidate ranking, genetic gain, and predictive accuracy and prediction bias.

**Results:**

Genotyping a subset of the study population provided insights into the level of relatedness in BSOs, allowing better genetic management. Due to the inbreeding detected within the genotyped provenances, we estimated genetic parameters both with and without accounting for inbreeding. The ssGBLUP model showed improved performance in terms of additive genetic variance and theoretical breeding value accuracy. Similarly, ssGBLUP showed improved predictive accuracy and lower bias than the pedigree-based relationship matrix (ABLUP).

**Conclusions:**

This study of *C. africana*, a species in decline due to deforestation and selective logging, revealed inbreeding depression. The provenance exhibiting the highest level of inbreeding had the poorest overall performance. The use of different relationship matrices and accounting for inbreeding did not substantially affect the ranking of candidate individuals. This is the first study of this approach in a tropical multipurpose tree species, and the analysed BSOs represent the primary effort to breed *C. africana*.

**Supplementary Information:**

The online version contains supplementary material available at 10.1186/s12864-023-09907-z.

## Background

In the tropical world, there is a need to undertake forest and landscape restoration to improve degraded former forest lands [1]. Combined with climate change, this poses huge challenges to both the amount and quality of the plant material used for this restoration. Consequently, there is a need for breeding and forest tree improvement of a multitude of species. Globally, large-scale reforestation and afforestation programmes have focused on a few genera and species, mainly conifers, such as *Pinus* spp. and *Picea* spp., or broadleaves, such as *Eucalyptus* spp. and *Acacia* spp., often involving intercontinental movement and monoculture plantations [2]. However, because of the risk of genetic diversity loss and the capacity to adapt to extreme climates, as well as to other ecological and socioeconomic issues [3], the use of multiple/diverse species seems to be a more realistic approach [4]. Conventional breeding is resource-intensive for managing and tracking the seed-generated families in the nursery and the progeny tests. Furthermore, in a conventional first-generation breeding programme, early selection is inevitably based on juvenile traits. This emphasises the importance of testing the possibility of using genomics in pedigree reconstruction and the estimation of additive relationships in natural populations of tropical tree species. This study considers the breeding of multipurpose tropical species of local/regional importance using genomic evaluations via a genotyping-by-sequencing approach, that is, DArT partial genome sequencing, which does not rely on the availability of genomic resources for the species.

Experimental plant genetics and breeding programmes rely on the ability to predict and visualise the inheritance of alleles underlying traits of interest [5]. To estimate genetic parameters (i.e., variance components and heritability), it is necessary to infer additive genetic relationships among individuals based on their known pedigrees [6]. Classical quantitative genetics uses this information on additive relationships to estimate heritability, covariance between traits, and genotype-by-environment interactions. In addition to enabling the estimation of genetic parameters, pedigree information is also critical for maintaining high genetic variation and low levels of inbreeding. These latter issues are critical for populations to face environmental changes and ensure long-term genetic gain [7]. However, there is a limitation related to the accuracy and completeness of the available pedigree information, especially in wild populations.

In the absence of pedigree information, molecular marker data have been utilised to reconstruct pedigrees since the 1970s and 1980s [8–11]. In forest trees, pedigree reconstruction via DNA markers and subsequent quantitative genetic analyses as a breeding approach was introduced in the 2000’s [12–15], but these first studies were based on using only a few highly variable microsatellite DNA markers. Despite the obvious potential for tree breeding via pedigree reconstruction, there are issues that need to be considered. One such issue is the incompleteness of sampling potential parents, as pedigree and/or sibship reconstruction based on a few DNA markers requires information on the parental population (see [16] for instance). This is particularly a challenge when one works with natural populations or with plantations established with commercial seed lots. In addition, even with successful pedigree or sib-ship reconstruction, there is a problem of hidden relatedness because the focus is only on one-generation relatedness, where each family is considered unrelated and no Mendelian sampling variance is considered. The accuracy of genetic parameter estimation and rankings of predicted breeding values can be affected by hidden relatedness [17–20].

Accurate estimation of relatedness between individuals in breeding populations using DNA markers is important not only to precisely estimate genetic parameters but also for effective inbreeding management [21]. Furthermore, DNA markers can assess the genetic diversity of the entire population across different gene pools [22].

*Cordia africana* is a fast-growing tree species that is highly valued in Ethiopia for its timber. As one of the most commercially utilised species, it plays an important role in generating household income from the sale of wood products [23]. In its current distribution, the populations of *C. africana* are heavily affected by deforestation, fragmentation, and selective logging. The northern part of Ethiopia has been extremely deforested. As a result, this species is mainly represented by scattered trees on farmlands, church compounds, and graveyards, while a relatively continuous forest only exists in a few spots [24, 25]. *Cordia africana* is an indigenous tree species that has been identified and given conservation priority nationwide in Ethiopia [26]. Moreover, *C. africana* is a priority species included in the tree breeding programme under the Provision of Adequate Tree Seed Portfolio (PATSPO), supporting afforestation efforts in Ethiopia [27]. This breeding programme of the *C. africana* is based on the establishment of breeding seedling orchards (BSOs). The improved seed produced by the BSOs is foreseen to play a significant role in species conservation, promoting its sustainable use by enabling the establishment of improved *C.africana* plantations for different end uses and thereby decreasing the pressure on natural populations. As the first generation of breeding this species, seeds were collected from the major growing areas of the country, with the aim of broadening the genetic basis and ensuring continuous gain.

The mating system of *C. africana* has not yet been specifically documented. However, as a long-living tree with an efficient means of pollen and seed dispersal, the species is speculated to be predominantly outcrossing [25]. Nonetheless, due to its hermaphrodite flowers [23], and no evidence of self-incompatibility, self-fertilisation might be possible under some conditions. Moreover, even in species that are not self-compatible, inbreeding can occur through mating between related individuals. As a species with a fragmented/scattered distribution and exposure to selective/illegal logging, the probability of building up co-ancestry is high. This makes it difficult to obtain a reliable estimate of genetic parameters using the conventional pedigree-based approach and increases the risk of selecting related materials. In recent years, the utilisation of realised genetic relationships has been shown to produce a more accurate estimation of genetic parameters by capturing within-family variation that arises from Mendelian segregation (e.g., [28]). This is because the method enables the detection of the realised genetic covariance based on a fraction of the genome that is identical by descent or by state between individuals [29]. However, since *C. africana* is not a model species and lacks genetic information, and because there are few resources allocated to the breeding programme, the expense of using large-scale genotyping in the operational breeding of this species is still not feasible. To overcome this, single-step genomic best linear unbiased prediction (ssGBLUP), an approach in which the realised genomic relatedness of a small portion of genotyped individuals is combined with a large proportion of non-genotyped individuals in a single genetic evaluation, has been proposed as an effective analytical method [30–32]. The ssGBLUP method combines the pedigree-based ***A***-matrix of non-genotyped individuals with the ***G***-matrix of genotyped individuals into a single genetic covariance hybrid ***H***-matrix. This method has been demonstrated to be an efficient option for improving the derived genetic parameters’ precision and breeding value accuracy from the actual generation (e.g., [33–35]) and for genomic prediction (e.g., [36–39]) in different tree species.

The aim of this study was to evaluate the efficiency of ssGBLUP, with minimum genotyping effort, in *C. africana* breeding. This study compared the results of ssGBLUP with pedigree-based best linear unbiased prediction (ABLUP) and genomic best linear unbiased prediction (GBLUP), based on various factors, including genetic parameters, theoretical accuracy of breeding values, selection candidate ranking, genetic gain, efficiency of the ssGBLUP method, and predictive accuracy and prediction bias. Additionally, by genotyping a subset of the study population, we aimed to determine the level of relatedness in the three BSOs and the potential effects of inbreeding on *C. africana*. This knowledge will be useful in better managing the genetic resources of *C. africana*. While previously mentioned studies have demonstrated the utility of ssGBLUP in tree breeding, this is the first report of the use of genomic tools in *C. africana* breeding. Finally, this is the first study of this approach in a tropical multipurpose tree species, and the analysed BSOs represent the primary effort to breed *C. africana*.

## Results

### Population and family structure

**Fig. 1:**
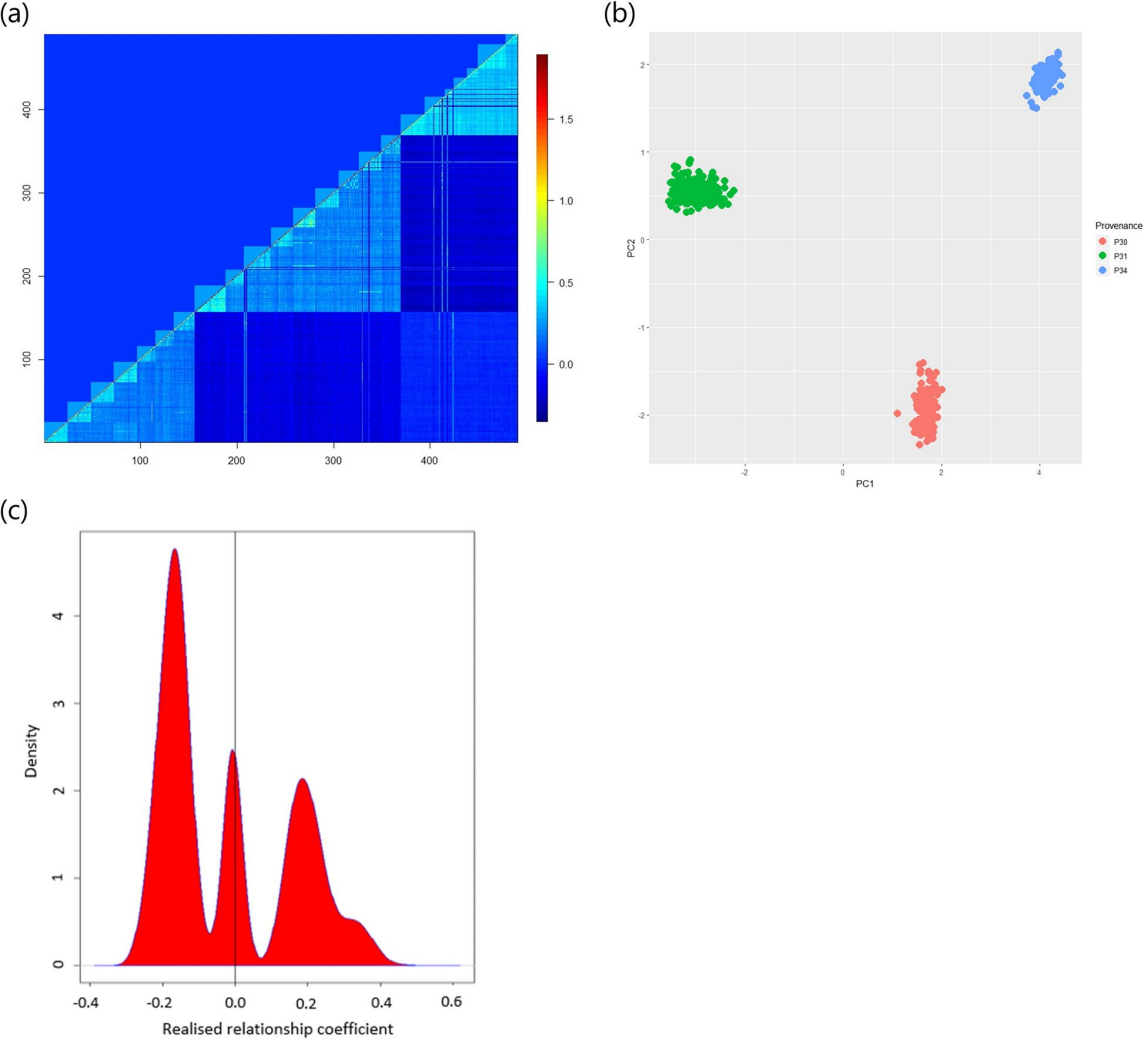
Heatmap of pairwise genomic relationship coefficients among the 490 genotyped *C. africana* trees (**a**), principal component analysis of the three genotyped provenances (**b**) and density plot of the four additive realised relationship coefficients within the *G*-matrix (**c**). In plot (**a**), the heat scale represents the degree of pairwise relationship coefficients for all pairs of trees. It is based on the provided pedigree (***A***-matrix before pedigree correction) in the upper diagonal and the realized genomic relationship coefficients (***G***-matrix) in the lower diagonal. The three light blue triangles are placed diagonally along the lower diagonal, indicating moderate relatedness between individuals within a provenance in the serial order of P30 (the first triangle), P31 (the second triangle), and P34 (the third triangle) from left to right. The smaller green boxes along the diagonal represent the families within the provenances based on the ***A***- and ***G***-matrix, on the upper and lower sides of the diagonal, respectively. The ***G***-matrix (lower diagonal) also shows pedigree errors in 15 individuals that appear to belong to different families, either within the same or different provenances. In plot (**b**), the first principal component (PC1) is plotted against the second principal component (PC2), and different colours represent different provenances: P30 in red, P31 in green, and P34 in blue. Plot (**c**) displays four peaks representing different levels of realised genetic relationships between individuals. The first peak (highest) shows the relationship between individuals from different provenances. The second peak represents the relationship between unrelated individuals among provenances and within provenances. The third peak represents the relationship between individuals from different families within the same provenance, and the fourth peak attached to the third peak represents the relationship within families

The pairwise relationship coefficients of the ***G***-matrix showed a clear provenance and family structure (Fig. 1a). The heatmap revealed separate grouping of the genotyped provenances, which coincided with the geographic distance between their origins (Supplementary Table S1). Provenance P30 included 161 trees from the North Bench zone (southern Ethiopia), provenance P31 included 212 trees from Adwa (northern Ethiopia), and provenance P34 included 121 trees from Harar (eastern Ethiopia). Similarly, principal component analysis of genotypes showed grouping of individuals according to these three provenances (Fig. 1b).

By examining the genomic pairwise relatedness among the 490 trees, we found various levels of genetic relationships within the population. The estimated relatedness between individuals, obtained by genetic marker analysis, did not always match the expected values, such as half siblings (expected value 0.25), and unrelated individuals (expected value 0.00). However, there was only little variation in these estimates across the different provenances. For the 490 trees, the average relatedness for unrelated individuals within provenance was above zero for most pairs and close to an expected half-sib relationship of 0.25 for many pairs that are assumed to be unrelated. The average minimum pairwise genomic relatedness between trees belonging to the same open-pollinated family was 0.28, while the average across all families in all three provenances was 0.39. In contrast, the genomic relatedness value between individuals from different provenances was negative for most pairs (Fig. 1c).

### Inbreeding and provenance performance

According to the diagonal elements of the ***G***-matrix, two of the three provenances showed an inbreeding coefficient *F*_*i*_ above zero. *F*_*i*_ was calculated by subtracting one from the diagonal elements of the ***G***-matrix [40]. Genotyped provenance P31 had the highest *F*_*i*_ value (0.54), while provenance P34 had the lowest (0.01). Provenance P30 had a *F*_*i*_ of 0.29. The average *F*_*i*_ across all genotyped provenances was 0.33. The best linear unbiased estimates (BLUE) from the ssGBLUP model of the genotyped provenances revealed that their height was consistent with their respective levels of inbreeding. Accordingly, P34 was highest, while P31 was lowest across all sites (Table 1).

**Table 1:** Provenance best linear unbiased estimates (BLUE) (and standard error) of height (in decimetres) and ranking within sites of the three genotyped provenances in the three breeding seed orchards

Provenance	ILRI		SM		Suba	
	**BLUE (decimetres)**	**Rank**	**BLUE ﻿(decimetres)**	**Rank**	**BLUE ﻿(decimetres)**	**Rank**
**P30**	16.0 (± 0.4)	2	9.2 (± 0.3)	2	12.7 (± 0.4)	2
**P31**	14.6 (± 0.3)	3	4.9 (± 0.3)	3	8.4 (± 0.3)	3
**P34**	17.9 (± 0.4)	1	11.1 (± 0.3)	1	13.5 (± 0.4)	1

*ILRI* International livestock research institute, *SM* Sekela Mariam, *Suba* Menagesha Suba, *BLUE* Best linear unbiased estimation

**Fig. 2:**
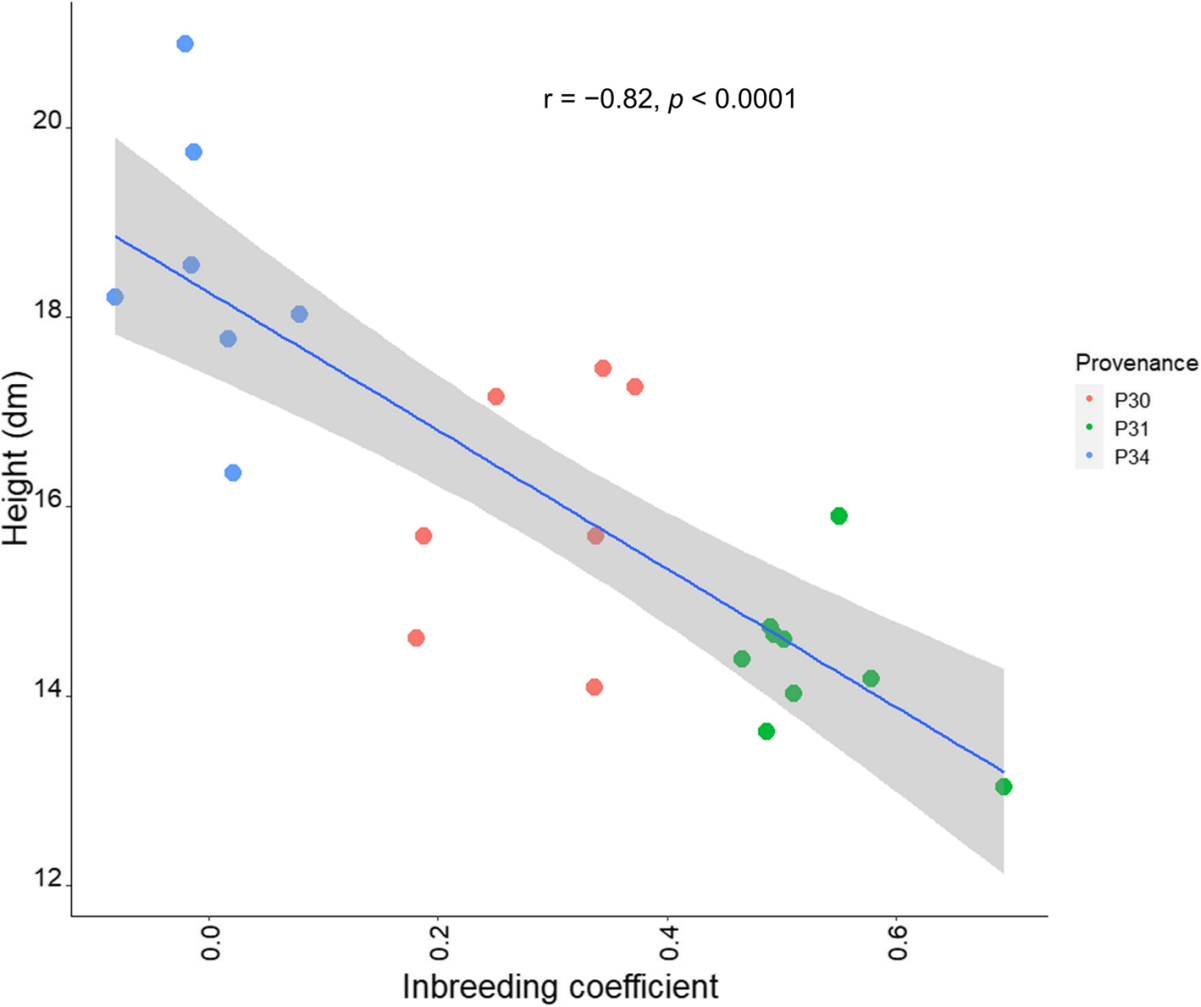
Correlation between the mean marker-based inbreeding coefficient and the mean height for 23 families representing the three provenances

To further examine the inbreeding effect, we conducted a correlation test between mean family height and mean family inbreeding coefficient. Overall, there was a strong indication of a negative effect of inbreeding on height (*r* =  − 0.82; Fig. 2).

### Additive genetic variances and heritability estimates

Without accounting for inbreeding, the additive variance estimates (${\sigma }_{a}^{2}$) for height using the ***A***-matrix (ABLUP-1) ranged from 2.32 in the SM site to 3.21 in the Suba site, while the estimates from the ssGBLUP-1 ranged from 2.63 in the ILRI site to 3.75 at Suba. When accounting for inbreeding, the additive variance using the ***A***-matrix (ABLUP-2) was reduced to 1.73 in ILRI, 1.52 in the SM site, and 2.24 in the Suba site (Table 2). The estimates from ssGBLUP-2 were 2.05 in ILRI, 2.04 in SM, and 2.93 in Suba. Although incorporating a selfing rate resulted in a considerable decrease in additive genetic variance and heritability in both the ABLUP and ssGBLUP models, the discrepancy was larger between the two ABLUP models than between the two ssGBLUP models. At the ILRI site, accounting for inbreeding decreased the additive genetic variance by 36.4% in ABLUP and 22.1% in ssGBLUP. In SM, accounting for inbreeding decreased the additive variance estimates by 34.5% and 23.9% in ABLUP and ssGBLUP, respectively. The Suba site showed the same pattern, with the estimate in ABLUP decreasing by 33.6% and the estimate in ssGBLUP decreasing by 21.9%. Generally, there was a constant increase in additive variance estimates when moving from ABLUP to ssGBLUP at all sites, except for the without-inbreeding scenario in the ILRI site, where it slightly decreased from 2.72 in ABLUP to 2.63 in ssGBLUP.

**Table 2:** Genetic parameter estimates for total height at different sites and using different models

**Sites**	**Model**	${\sigma }_{a}^{2}$(± SE)	${\sigma }_{b}^{2}$(± SE)	${\sigma }_{e}^{2}$(± SE)	${h}^{2}$(± SE)
**ILRI**	**ABLUP-1**	2.72 (0.73)	1.41 (0.54)	6.50 (0.66)	0.26 (0.07)
	**ssGBLUP-1**	2.63 (0.63)	1.41 (0.54)	6.66 (0.57)	0.25 (0.06)
	**ABLUP-2**	1.73 (0.48)	1.41 (0.54)	7.34 (0.47)	0.17 (0.05)
	**ssGBLUP-2**	2.05 (0.53)	1.41 (0.54)	7.11 (0.50)	0.19 (0.05)
	**GBLUP**	2.22 (0.97)	-	6.71 (0.81)	0.25 (0.24)
**SM**	**ABLUP-1**	2.32 (0.61)	2.90 (0.98)	6.85 (0.56)	0.19 (0.05)
	**ssGBLUP-1**	2.68 (0.68)	2.90 (0.98)	6.55 (0.62)	0.22 (0.06)
	**ABLUP-2**	1.52 (0.41)	2.90 (0.98)	7.53 (0.41)	0.13 (0.04)
	**ssGBLUP-2**	2.04 (0.53)	2.90 (0.98)	7.10 (0.50)	0.17 (0.04)
	**GBLUP**	-	-	-	-
**Suba**	**ABLUP-1**	3.21 (0.85)	1.60 (0.65)	5.29 (0.75)	0.32 (0.08)
	**ssGBLUP-1**	3.75 (0.96)	1.60 (0.65)	4.86 (0.84)	0.37 (0.09)
	**ABLUP-2**	2.13 (0.59)	1.60 (0.65)	6.20 (0.55)	0.21 (0.06)
	**ssGBLUP-2**	2.93 (0.76)	1.60 (0.65)	5.58 (0.68)	0.29 (0.07)
	**GBLUP**	-	-	-	-

*ILRI* International livestock research institute, *SM* Sekela Mariam, *Suba* Menagesha Suba, *ABLUP-1* Pedigree-based ABLUP model without accounting for inbreeding, *ABLUP-2* Pedigree-based ABLUP model accounting for inbreeding, *ssGBLUP-1* Genomic-based ssGBLUP model without accounting for inbreeding, *ssGBLUP-2* Genomic-based ssGBLUP model accounting for inbreeding, *GBLUP* Genomic best linear unbiased prediction, ${\sigma }_{a}^{2}$ Additive variance, ${\sigma }_{b}^{2}$ Bulk (seed-lot) variance, ${\sigma }_{e}^{2}$ Residual variances, ${h}^{2}$ Narrow-sense heritability

As expected, the narrow-sense heritability estimates of the ABLUP and ssGBLUP models mirrored those of the additive genetic variance estimates in both scenarios at all sites. The highest estimates were observed for the Suba site in all models studied compared to the same models at the other sites. Generally, ssGBLUP showed an improved heritability compared to its counterpart ABLUP within the same scenario, except for the scenario without inbreeding at the ILRI site, where ABLUP showed a slightly higher estimate (0.26) compared to ssGBLUP (0.25) (Table 2).

### Additive genetic correlations across sites

Additive genetic correlations between sites from all multiple-site models varied from 0.40 to 0.53 (see Supplementary Fig. S1 for details). ILRI and SM showed a slightly higher correlation in all models. The lowest average across-model genetic correlation between sites (0.44) was observed between Suba and ILRI. The moderate genetic correlations between sites indicates the presence of a genotype-by-environment interaction.

### Theoretical accuracy of breeding values

Overall, the highest average theoretical accuracy of the predicted breeding values was observed for ssGBLUP-1 in Suba (0.68 ± 0.02), followed by GBLUP for genotyped individuals in ILRI (0.65 ± 0.04) (Table 3). The use of genomic relatedness did not only improve the accuracy of breeding values for trees from genotyped families but also for trees from families with no genotyped individuals. In the inbreeding scenario, the genotyped site IRLI showed that ssGBLUP-2 improved the breeding value accuracy by 12% compared to ABLUP-2. This increment was higher for SM (17%) and Suba (16.4%) sites. The “without inbreeding” scenario showed the same pattern, except for the ILRI site, where theoretical accuracy decreased marginally by 1.7%. As expected, given the higher estimates of the additive genetic variances (Table 2), the ABLUP and ssGBLUP models without inbreeding showed higher theoretical accuracies than the respective models “with inbreeding”.

**Table 3:** Mean and standard deviations of estimated theoretical accuracy of the prediction of breeding values for ABLUP and ssGBLUP models with (ABLUP-2 and ssGBLUP-2) and without accounting for inbreeding (ABLUP-1 and ssGBLUP-1) across the three investigated sites

Model	Sites		
	**ILRI**	**SM**	**Suba**
**ABLUP-1**	0.59 (± 0.04)	0.55 (± 0.05)	0.64 (± 0.03)
**ssGBLUP-1**	0.58 (± 0.04)	0.58 (± 0.04)	0.68 (± 0.02)
**ABLUP-2**	0.50 (± 0.06)	0.47 (± 0.06)	0.55 (± 0.04)
**ssGBLUP-2**	0.56 (± 0.07)	0.55 (± 0.07)	0.64 (± 0.05)
**GBLUP**	0.65 (± 0.04)	-	-

*ABLUP-1* Pedigree-based ABLUP model without accounting for inbreeding, *ABLUP-2* Pedigree-based ABLUP model accounting for inbreeding, *ssGBLUP-1* Genomic-based ssGBLUP model without accounting for inbreeding, *ssGBLUP-2* Genomic-based ssGBLUP model accounting for inbreeding, *GBLUP* Genomic best linear unbiased prediction, *ILRI* International livestock research institute, *SM* Sekela Mariam, *Suba* Menagesha Suba

### Efficiency

Efficiency ($E$) in the accuracy of predicted breeding values, i.e., the proportion of extra benefit obtained from using ssGBLUP calculated for the genotyped site (ILRI) [41], was 0.4 for the inbreeding scenarios. Thus, assuming that GBLUP was 100% more efficient than ABLUP, ssGBLUP was 40% more efficient than ABLUP when inbreeding was considered. However, in the scenario without inbreeding, due to the lower theoretical accuracy of the breeding values (because of lower additive genetic variance) of ssGBLUP at the ILRI site, ssGBLUP had no advantage over ABLUP.

### Candidate ranking and expected genetic gain

The proportion of common selection candidates in the top 10% of trees was used to test the impact of including genomic information and inbreeding in the prediction of breeding values. The lowest proportion of common candidates (96.2%) was observed between the ABLUP-1 (without inbreeding) and ssGBLUP-2 (with inbreeding) models. The two ssGBLUP models showed 96.6% common candidates. The two ABLUP models showed a similar proportion of shared candidates (96.5%). The ABLUP and ssGBLUP models without considering inbreeding (i.e., ABLUP-1 vs. ssGBLUP-1) showed a large proportion of common candidates (98.2%), followed by the same models in the inbreeding scenario (97.9%). Thus, neither the change in the model nor accounting for inbreeding had a substantial effect on the ranking of the top 10% of candidates. However, there were still some changes in the ranking of individuals. Supplementary Fig. S2 shows the change in ranks for the top 50 individuals between the models.

The proportion of selected candidates from different provenances in the top 10% was used to determine the impact of inbreeding (Table 4). The provenance with the highest inbreeding (P31, *F*_*i*_ = 0.54) had the lowest proportion of selection candidates in the top 10% (T10%, Table 4), while the provenance with the least inbreeding (P34, *F*_*i*_ = 0.01) had the highest proportion of selection candidates. To further check for signs of inbreeding depression, we also examined the proportion of the genotyped provenances among the worst-performing individuals (in the lowest 10% of breeding values) (Table 4). In all models, we found that over 98% of the low-ranked individuals came from the provenance with the highest level of inbreeding (P31).

**Table 4:** Proportion of individuals in the top and bottom 10% of individuals (in percentage) from the three genotyped provenances for the four models evaluated. The remaining trees in the top- and bottom-ranked individuals are from the bulk seed lots

Provenances	ABLUP-1		ssGBLUP-1		ABLUP-2		ssGBLUP-2	
	**T10%**	**L10%**	**T10%**	**L10%**	**T10%**	**L10%**	**T10%**	**L10%**
**P30**	22.6	0.0	22.5	0.0	23.7	0.0	24.4	0.0
**P31**	12.2	98.7	11.8	98.0	11.1	99.9	9.8	99.5
**P34**	32.8	0.0	33.6	0.0	33.0	0.0	33.4	0.0

*ABLUP-1* Pedigree-based ABLUP model without accounting for inbreeding, *ABLUP-2* Pedigree-based ABLUP model accounting for inbreeding, *ssGBLUP-1* Genomic-based ssGBLUP model without accounting for inbreeding, *ssGBLUP-2* Genomic-based ssGBLUP model accounting for inbreeding, *T10%* Top 10% ranked selection candidates), *L10%* Lowest 10% selection candidates

Finally, the expected genetic gains from the top-ranked 10% were 57.5% and 57.6% for ABLUP-1 and ssGBLUP-1, respectively. The models in the inbreeding scenario showed slightly lower genetic gain, i.e., 55.7% for ABLUP-2 and 56.8% for ssGBLUP-2, over the original population mean.

### Predictive accuracy and prediction bias of the models

**Fig. 3:**
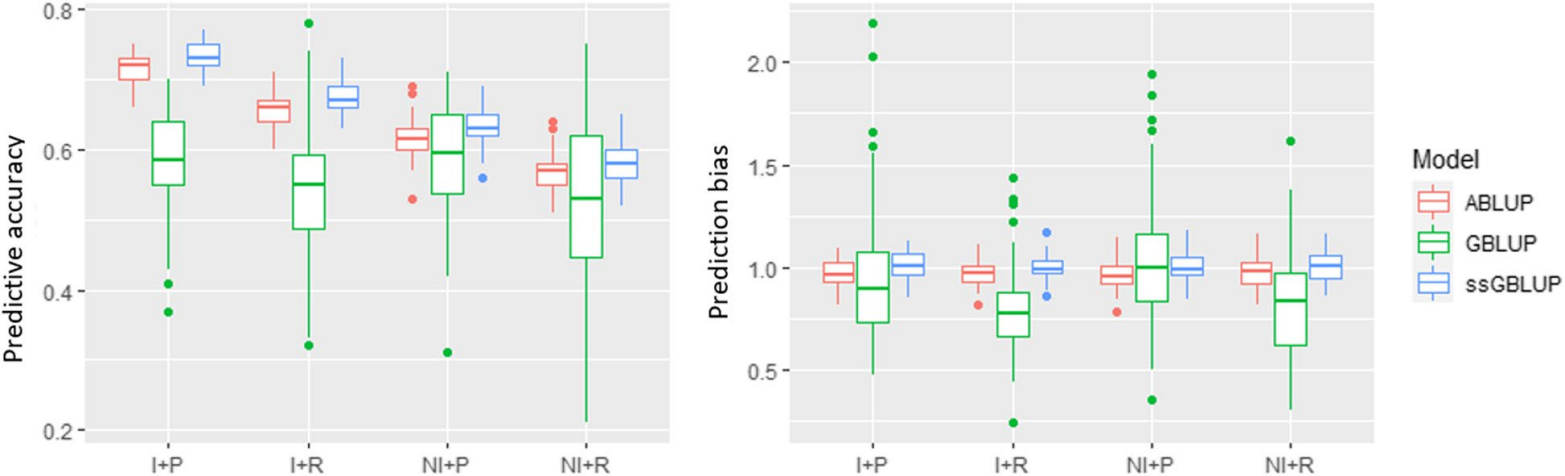
Average predictive accuracy (PA) and prediction bias (PB) for the ABLUP, GBLUP, and ssGBLUP prediction models studied using the four combined scenarios. These scenarios included the presence (I) or absence (NI) of inbreeding and random (R) and within-provenance (P) cross-validation scenarios. NOTE: I + P = Model with inbreeding combined with within provenance cross-validation; I + R = Model with inbreeding combined with random cross-validation; NI + P = Model without inbreeding combined with within provenance cross-validation; NI + R = Model without inbreeding combined with random cross-validation

Overall, the ssGBLUP prediction models showed the highest predictive accuracy (PA) compared to the ABLUP and GBLUP models for all studied scenarios (i.e., with and without inbreeding, and random and within provenance cross-validation). From the two cross-validation scenarios (i.e., random and within provenance), the PA obtained from within provenance cross-validation was higher than the PA obtained from random cross-validation (Fig. 3). Overall, the inbreeding scenario showed a higher PA than non-inbreeding. In this sense, the ssGBLUP model in the inbreeding scenario (ssGBLUP-2) combined with within-provenance sampling showed the highest predictive accuracy (0.73 ± 0.02) (Supplementary Table S2), with almost similar outcome as the ABLUP model in the same scenario (ABLUP-2, 0.72 ± 0.02). Meanwhile, the lowest PA was observed for the GBLUP model in random cross-validation (0.53 ± 0.11). In all cases, both the ssGBLUP and ABLUP models showed a lower prediction bias (a value of ~ 1), while the GBLUP model had the highest bias in random cross-validation scenarios (0.79 ± 0.23). This might be attributed to the relatively small number of trees in the training and validation population (490 trees), which could have affected the model’s performance.

## Discussion

To ensure increased resilience of managed production plantations under climate change, it is crucial to plant tested forest reproductive material [42]. The availability of improved material serves a dual purpose; meeting the demand for timber and wood products but also relieving the pressure on the natural forests and helping restore locally endangered species. This is particularly true for *C. africana* in Ethiopia, where the habitats and populations are severely affected by deforestation, fragmentation, and selective logging. Thus, overcoming the deforestation issue while meeting the local demand for timber and wood products requires a supply of genetically diverse, healthy, and productive material. In this regard, the use of quantitative genetics methodology in forest tree breeding is widely recognised for its ability to deliver results [43]. However, there is limited time and resources to start traditional long-term breeding programmes to genetically improve the species. Incorporating genomic tools into practical tree breeding has been reported to increase gain by reducing the breeding cycle and improving selection efficiency [44].

This study tested the efficiency of breeding *C. africana* with the ssGBLUP approach using minimum genotyping effort and compared it to the ABLUP and GBLUP approaches. Comparisons included additive genetic variance, accuracy of the predicted breeding values, ranking of candidates for selection, genetic gain, efficiency of the ssGBLUP approach, and predictive accuracy and prediction bias. In addition, genotyping a subset of the study population provided insight into the degree of relatedness within the BSOs. This enabled evaluation of the potential impact of inbreeding in *C. africana* and enhanced our understanding of relatedness in the BSOs, which is useful for genetic management. This may imply possible restrictions on selection to avoid significant decrease in genetic variation. Restrictions that would otherwise not be implemented due to a lack of knowledge of relatedness in the material.

The pairwise genomic relationship coefficients of the *C. africana* genotyped trees differed from the expected pedigree-based values for half-sib and unrelated individuals. However, there was a clear differentiation among provenances, as individuals from different provenances appeared to be unrelated. The increased pairwise half-sib values (i.e., expected values 0.25 vs. realised values averaged across families 0.39) could be due to: 1) the build-up of co-ancestry and gene flow restrictions resulting from fragmented distribution of the species due to exposure to selective logging; and 2) background inbreeding due to lack of sufficient genetic separation, primarily due to close distance between the selected mother trees. This means that assuming an inbreeding level of zero, as is done in the classical breeding approach, could lead to an overestimation of genetic parameters, i.e., additive variance and heritability, and underestimation of relatedness across the studied sites. In this study, accounting for inbreeding decreased the additive variance estimate and heritability. Inflated heritability estimates without proper consideration of inbreeding concur with the results of other studies [45]. Moreover, hidden relatedness has been reported to result in upwardly biased estimates of heritability caused by the overestimation of additive genetic variance due to the unrealistic assumption of pure half-sibling relatedness within open-pollinated families, as well as the absence of historical relatedness among the parents [17, 46, 21].

In our study, the ssGBLUP models showed a constant increase in additive variance estimates compared to the ABLUP models at all sites, except for the “without inbreeding” scenario at the ILRI site. This is in line with results reported for growth traits in other tree species, for example, *Eucalyptus* [47, 48]*,* lodgepole pine [38], white spruce [49], and loblolly pine [50]. However, studies on other tree species have reported that models using genomic evaluation (GBLUP or ssGBLUP) show decreased or similar additive variance estimates compared to models using pedigree-based information only for growth and wood quality traits [51–53]. The difference in genetic variance estimates between the pedigree and genomic-based relationship matrix could be because pedigree and genomic-based relationship matrices pertain to separate base populations, with genomic relationships reflecting the genotyped population and pedigree relationships reflecting the founders of the population under study [54].

The ssGBLUP models have an advantage over the ABLUP models in that they use the realised genomic relationship among individuals, whereas the ABLUP models are completely dependent on the assumed pedigree structure created by mating designs. The accuracy of the predicted breeding values is of great importance to tree breeders, and improvement in accuracy can be achieved by using genetic markers in the evaluation [33]. Here, the GBLUP showed the highest mean theoretical accuracy for breeding values. In all cases, the mean breeding value accuracy increased from ABLUP to ssGBLUP across the investigated genotyped and non-genotyped sites. Similar findings were reported in studies of forest trees (e.g., [34, 35, 37, 55]), mostly because of a smaller prediction error variance in ssGBLUP.

The genetic architecture of the trait, the choice of model, and the density of available markers in the model are factors that affect the accuracy of genomic prediction [56]. The use of genetic markers, capturing the additive relatedness among individuals, also captures the linkage disequilibrium (LD) between the SNPs and quantitative trait loci (QTLs), which affects the accuracy of the genomic estimated breeding values [57, 58]. The ssGBLUP provides a useful framework for combining the DNA marker data of the genotyped portion of the test population with the ***A***-matrix used in BLUP analyses. As a result, it serves as a compromise between ABLUP and GBLUP [38]. In both tested cross-validation scenarios, i.e., random and within provenance cross-validation, the ABLUP and ssGBLUP models showed high PA and low PB, while GBLUP showed the lowest PA and highest PB. The reason for the lowest PA and higher PB in GBLUP could be because only 6.1 percent of the entire study population was genotyped. Consequently, we had substantially smaller training and validation sets. As previously observed in other forest trees using simulation [59] and empirical datasets [47, 60], the PA improved with the increasing size of the training set [39]. The within-provenance cross-validation scenarios showed a slightly higher average PA for the three predictive models studied. The three provenances in this study appeared to be unrelated. Moreover, our results showed that models in which inbreeding was considered showed improved PA. Increased relatedness between the training and validation populations is known to increase the model’s PA [61]. Thus, improved PA in the case of both within-provenance cross-validation and inbreeding scenarios could be attributed to the higher relatedness between the training and validation populations. Genetic diversity within the training population has also been reported to influence PA [62, 63].

Genetic diversity management is an integral part of tree breeding [64]. Retaining genetic diversity is crucial for long-term genetic gain, and also limiting inbreeding and hence inbreeding depression [65, 66]. Currently, *C. africana* populations in Ethiopia are severely affected by deforestation, fragmentation, and selective logging. Forest loss and fragmentation are known to change the landscape’s connectedness and composition [67]. A few examples of how these changes may impact genetic diversity include instances linked to decreased population sizes and the isolation of residual populations, which can affect and limit gene flow and cause the direct loss of genes [68]. The BSOs in this study were established by taking the necessary precautions to avoid inbreeding, i.e., by selecting mother trees with a minimum of 100 m distance from each other and in areas with a larger number of trees when possible. However, since the selfing rate might vary among species, populations, and individuals, it should be estimated using DNA marker data from the population under study [48]. This is especially true for *C. africana* in Ethiopia, where the fragmentation makes diversity management tricky. This is evident from the variation in the level of inbreeding between the genotyped provenances, with a mean inbreeding level of 0.33. Although it is difficult to conclude that *C. africana* suffers from inbreeding depression based on limited provenances, the most inbred provenance (P31, *Fi* = 0.54) showed signs of inbreeding depression, with the lowest overall performance. It also had the lowest proportion of selection candidates in the top 10%. In contrast, the least inbred provenance (P34, *Fi* = 0.01) showed the best overall performance. Similarly, the correlation between height and inbreeding at the family level (r =  − 0.82, *p* < 0.0001) was a strong indicator of the negative influence of inbreeding. However, there are possible confounding effects of provenances and inbreeding, and the correlation between height and the inbreeding coefficient seems weaker and inconclusive within provenances. This could be due to the relatively small differences in the inbreeding coefficients within provenances.

A comparison of breeding value rankings from different models revealed change in ranks between the ABLUP and ssGBLUP models. Nevertheless, all models shared most of their top 10% of trees. The lowest proportion of common candidates in the top 10% of trees was between ABLUP-1 and ssGBLUP-2 (96.2%). This was expected because these two models represent scenarios without any genetic information and scenarios with all genetic information available. This maximises the information gap between the ABLUP-1 and ssGBLUP-2 models, resulting in a lower proportion of common selection candidates. The proportion of common candidates between the two ABLUP models with or without considering inbreeding was 96.5%, indicating that accounting for inbreeding has a minimal impact on the ranking of selection candidates. Generally, although the 10% selection scenarios using different approaches largely showed common candidate trees, this could, to some extent, be due to high inbreeding levels and depression. Differences in selected candidates between ABLUP-1 and ssGBLUP-2 would likely have been higher in the case of lower inbreeding without inbreeding depression. This means that the gap in the inbreeding level between provenances and the resulting impact on individual performance contributed to the selection of similar candidates across the models.

This study stresses the importance of sampling from multiple populations of native tree species, such as *C. africana*, having small, scattered populations with discontinuous tree distributions to enhance genetic diversity in BSOs. This is further emphasised because of the genotype-by-environment interactions indicated by moderate genetic correlations across the BSOs, despite being situated at almost similar altitudes. The differential performance of the species at the three BSOs could be attributed to the site differences in terms of soil nutrient, windiness, and other macro- and micro-climatic conditions. This species tends to struggle to grow in windy areas. Thus, the fact that Suba and SM sites are located on hilly sites could be the reason for the relatively slow early growth at these sites compared to ILRI.

Only 33.8% of the families had genotyped individuals. Genotyping individuals from all families could potentially have additional benefits for ssGBLUP models. Under fixed costs, this can be achieved by decreasing the number of individuals per family. We suggest conducting an in-depth population genetic analysis with more provenances to confirm whether the inbreeding level observed in the study is representative of *C. africana* populations in the country. Moreover, understanding the current genetic diversity of the species is crucial in devising appropriate measures for its conservation and sustainable utilisation.

## Conclusion(s)

We tested the efficiency of ssGBLUP with minimum genotyping effort in genetic management and breeding of *C. africana* in BSOs. Following the inbreeding detected in two of the three genotyped provenances, we compared the ABLUP and ssGBLUP models with and without accounting for inbreeding. Although both evaluation models, with or without accounting for inbreeding, had similar top 10% selection candidates, the ssGBLUP model displayed better performance in terms of additive genetic variance, theoretical breeding value accuracy, predictive accuracy, and prediction bias. Therefore, the ssGBLUP model was more reliable and accurate in determining breeding values, and as a result, the likelihood of correctly ranking selection candidates was higher. The inbreeding problem detected in two of the three genotyped provenances could potentially be broken in the next generation due to mating among the genetically differentiated provenances. 

## Methods

### Description of breeding seedling orchards (BSOs) and genetic material

The *C. africana* BSOs in this study were established at three sites in Ethiopia: Sekela Mariam Forest (SM), International Livestock Research Institute Ethiopia campus (ILRI), and Menagesha Suba Forest (Suba), as part of PATSPO, a national tree seed project in Ethiopia. These BSOs serve as test sites for *C. africana* germplasm, with the objective of developing an improved seed source through selection. The field experimental design for all locations was a randomised block design with single-tree plots.

The genetic material used in this study was obtained from 63 open-pollinated families from three native stands/provenances in Ethiopia: North Bench (P30), Adwa (P31), and Harar (P34). The number of families by provenance ranged from 20 to 23. Furthermore, bulk collections from 25 provenances lacking family information were also included. Details of the BSOs are summarised in Table 5.

**Table 5:** Description of the three *Cordia africana* breeding seedling orchards used in the study

Breeding Seedling Orchard	ILRI	SM	Suba
**Location**	International Livestock Research Institute, Ethiopia campus	Sekela Mariam Forest	Menagesha Suba Forest
**Coordinates**	9°0′50'' N 38°48′55'' E	10°35′52'' N 37°29′20'' E	8°57′18'' N 38°31′58'' E
**Altitude (masl)**	2351–2358	2420–2433	2290–2329
**Previous land use**	Not in use	Plantation forest	Plantation forest
**Planting date**	August 2018	August 2018	August 2018
**Plant origin**	Seed	Seed	Seed
**Number of initial trees**	2633	3600	2040
**Number of families**	53	61	55
**Number of bulk seed lots**	18	25	18
**Number of provenances**	3	3	3
**Block**	22	30	17
**Plot**	Single tree	Single tree	Single tree
**Spacing (m)**	2 × 2	2 × 2	2 × 2
**Number of phenotyped trees**	2600	3519	1951

*masl* Metres above sea level, *m* Metre

### Phenotyping and leaf sample collection

The height data (in decimetres) were collected from the three BSOs two years after planting. Families with high survival rate allowed for the sampling of an adequate number of individuals within the family, while also representing three main provenances in the country; thus, they were selected for SNP genotyping. Supplementary Table S3 provides details on the number of trees with phenotypic data and the number of trees sampled per genotyped family in each provenance. Leaf samples were collected from the ILRI BSO, silica gel dried in zip-lock bags for DNA extraction.

### DNA extraction, genotyping, and data preprocessing

DNA was extracted with the DNeasy Plant Mini Kit from QIAGEN (Germany). The DNA samples were sent to Diversity Arrays Technology (DArT) (https://www.diversityarrays.com) in Canberra, Australia, for DArTSeq genotyping.

The DArTseq genotyping [69] resulted in 9591 raw SNPs in the 550 genotyped individuals. Missing data across each locus were calculated, and loci with missing data in 15% of the trees/individuals were excluded. The heterozygosity per locus was used as a further filtering criterion, in which loci with ≤ 0.05 heterozygosity were removed. The filtering of data was also done across samples, and individuals with missing data in 15% of the loci were excluded. These filtrations reduced the SNPs and individuals to 4373 and 526, respectively. Filtering was done using the qc.filtering function in the R package ASRgenomics [70].

### Pedigree correction

Using the filtered SNPs, we validated and corrected the pedigree of the open-pollinated families based on a comparison of the expected versus observed additive genetic relationships. From the ***G***-matrix estimated following VanRaden [29], we examined the samples’ pairwise additive relationship coefficients for large deviations from their expected values, and the correct mother was manually reassigned. Thirty-six trees were removed for parent conflict. Of the remaining 490 trees, the pedigree records were corrected for 15 trees. The final set of genotyped individuals ranged from 7 to 32 trees per family. Principal component analysis of the ***G***-matrix was done to reveal potential grouping of the genotyped provenances. Supplementary Table S4 displays the number of trees analysed after correcting their pedigree and excluding trees with conflicting family information. Overall, 8070 trees were used for the analysis.

### Statistical analysis

Due to spatial heterogeneity within the BSOs, and for computational efficiency, the statistical analysis was conducted in two stages following Cappa et al. [39]. First, single-site analyses were made using a pedigree-based classical a priori design model, and an a posteriori spatial model with a first-order autoregressive error (co)variance structure (AR1 × AR1) [36]. The following single-trait single-site pedigree-based individual-tree mixed model was used:

1 $${\varvec{y}}={\varvec{X}}{\varvec{\beta}}+{{\varvec{Z}}}_{b}{\varvec{b}}+ {{\varvec{Z}}}_{a}{\varvec{a}}+{\varvec{e}}$$

where ${\varvec{y}}$ is the vector of phenotypic data; ${\varvec{\beta}}$ is the vector of fixed effects for blocks and provenances; ${\varvec{b}}$ is the vector of random bulk seed lot effects; ${\varvec{a}}$ is the vector of random effects that represents additive genetic effects (or breeding values), following a normal distribution with zero mean and covariance matrix ***A*** ${\sigma }_{a}^{2}$, where ***A*** is the average numerator relationship matrix derived from the pedigree information [71] and ${\sigma }_{a}^{2}$ is the additive genetic variance; and ${\varvec{e}}$ is the vector of the random residual effects following a normal distribution with zero mean and (co)variance matrix ${{\varvec{I}}\sigma }_{e}^{2}$, where ${\varvec{I}}$ is the identity matrix and ${\sigma }_{e}^{2}$ is the residual error variance. For the spatial autoregressive model, vector ${\varvec{e}}$ was partitioned into spatially dependent (ξ) and spatially independent (η) residuals. Therefore, the residual (co)variance matrix can be expressed as ${\sigma }_{\upxi }^{2}\left[AR1\left({\uprho }_{col}\right) \otimes AR1\left({\uprho }_{row}\right)\right]+ {\sigma }_{\upeta }^{2}{\varvec{I}}$, where ${\sigma }_{\upxi }^{2}$ is the spatially dependent variance; ${\sigma }_{\upeta }^{2}$ is the spatially independent variance; $AR1$(ρ) is the first-order autoregressive correlation process; ${\uprho }_{col}$ and ${\uprho }_{row}$ are autocorrelations parameters for columns and rows, respectively; and ⊗ denotes the Kronecker product. ${\varvec{X}}$*,* ${{\varvec{Z}}}_{b}$, and ${{\varvec{Z}}}_{a}$*,* are incidence matrices relating fixed and random effects to measurements in vector ${\varvec{y}}$*.*

For individuals from bulk seed lots, an extra independent variance was fitted. This approach was used to avoid bias in estimates of additive genetic variances by the bulk trees, as they were considered unrelated when their actual relationship was unknown [72].

Finally, the adjusted phenotype data were generated by subtracting the estimated block and the autoregressive residual effects from the corresponding raw phenotypes.

In the second stage, the adjusted phenotypes were analysed using the following pedigree-based (ABLUP) multiple-site individual-tree mixed model:

2 $$\left[\begin{array}{c}{{\varvec{y}}}_{1}\\ {{\varvec{y}}}_{2}\\ {{\varvec{y}}}_{3}\end{array}\right]= \left[\begin{array}{ccc}{{\varvec{X}}}_{1}& 0& 0\\ 0& {{\varvec{X}}}_{2}& 0\\ 0& 0& {{\varvec{X}}}_{3}\end{array}\right]\left[\begin{array}{c}{{\varvec{\beta}}}_{1}\\ {{\varvec{\beta}}}_{2}\\ {{\varvec{\beta}}}_{3}\end{array}\right]+\left[\begin{array}{ccc}{{\varvec{Z}}}_{{b}_{1}}& 0& 0\\ 0& {{\varvec{Z}}}_{{b}_{2}}& 0\\ 0& 0& {{\varvec{Z}}}_{{b}_{3}}\end{array}\right]\left[\begin{array}{c}{{\varvec{b}}}_{1}\\ {{\varvec{b}}}_{2}\\ {{\varvec{b}}}_{3}\end{array}\right]+\left[\begin{array}{ccc}{{\varvec{Z}}}_{{a}_{1}}& 0& 0\\ 0& {{\varvec{Z}}}_{{a}_{2}}& 0\\ 0& 0& {{\varvec{Z}}}_{{a}_{3}}\end{array}\right]\left[\begin{array}{c}{{\varvec{a}}}_{1}\\ {{\varvec{a}}}_{2}\\ {{\varvec{a}}}_{3}\end{array}\right]+ \left[\begin{array}{c}{{\varvec{e}}}_{1}\\ {{\varvec{e}}}_{2}\\ {{\varvec{e}}}_{3}\end{array}\right]$$

where ${\varvec{y}}=\boldsymbol{ }\left[{{\varvec{y}}}_{1}^{\mathrm{^{\prime}}},{{\varvec{y}}}_{2}^{\mathrm{^{\prime}}},{{\varvec{y}}}_{3}^{\mathrm{^{\prime}}}\right]$ is the vector of individual tree adjusted-phenotypes for the sites (1 = ILRI, 2 = SM, and 3 = Suba); ${\varvec{\upbeta}}= \left[{{\varvec{\upbeta}}}_{1}^{\mathrm{^{\prime}}}, {{{\varvec{\upbeta}}}_{2}^{\mathrm{^{\prime}}},{\varvec{\upbeta}}}_{3}^{\mathrm{^{\prime}}}\right]$ is the vector of fixed effects of provenance for each site; ${\varvec{b}}= \left[{{\varvec{b}}}_{1}^{\mathrm{^{\prime}}}, {{{\varvec{b}}}_{2}^{\mathrm{^{\prime}}},{\varvec{b}}}_{3}^{\mathrm{^{\prime}}}\right]$ is the vector of random bulk seed lot effects for each site distributed as ${\varvec{b}}\sim {\varvec{N}}\left(0,{\boldsymbol{\Sigma }}_{{\varvec{b}}}\otimes{\varvec{I}}\right)$, where ${\boldsymbol{\Sigma }}_{{\varvec{b}}}$ is the (co)variance of the bulk seed lot effects; and ${\varvec{a}}= \left[{{\varvec{a}}}_{1}^{\mathrm{^{\prime}}}, {{{\varvec{a}}}_{2}^{\mathrm{^{\prime}}},{\varvec{a}}}_{3}^{\mathrm{^{\prime}}}\right]$ is the vector of additive genetic effects random vector distributed as ${\varvec{a}}\sim {\varvec{N}}\left(0,{\boldsymbol{\Sigma }}_{{\varvec{a}}}\otimes{\varvec{A}}\right)$, where ${\boldsymbol{\Sigma }}_{{\varvec{a}}}$ is the unstructured genetic effects (co)variance matrix between sites and ${\varvec{A}}$ is defined above;. Finally, ${\varvec{e}}= \left[{{\varvec{e}}}_{1}^{\mathrm{^{\prime}}}, {{\varvec{e}}}_{2}^{\mathrm{^{\prime}}}, {{\varvec{e}}}_{3}^{\mathrm{^{\prime}}}\right]$ is the vector of random residuals distributed as ${\varvec{e}}\sim {\varvec{N}}(0,{{\varvec{R}}}_{0}\otimes{\varvec{I}})$, where ${{\varvec{R}}}_{0}$ is the residual (co)variance matrix for the three sites with dimension 3 × 3. We assumed an unstructured (co)variance matrix for the genetic and bulk seed lot effects (${\boldsymbol{\Sigma }}_{{\varvec{a}}}$ and ${\boldsymbol{\Sigma }}_{{\varvec{b}}}$, respectively). The matrices ${{\varvec{X}}}_{1}$, ${{\varvec{X}}}_{2}$, and ${{\varvec{X}}}_{3}$ and ${{\varvec{Z}}}_{{a}_{1}}$, ${{\varvec{Z}}}_{{a}_{2}}$, and ${{\varvec{Z}}}_{{a}_{3}}$ relate the observation to the means of the provenance effects in ${\varvec{\upbeta}}$ and the additive genetic effects for each tree in ${\varvec{a}}$. The symbol “´”, indicates the transpose operation.

The average-numerator relationship ***A***-matrix was computed using the corrected pedigree data. In addition, a modified numerator relationship matrix (***A***_2_) that considers partial selfing was computed according to Dutkowski et al. [73]. Both matrices were created in the ASReml-R version 4.0 [74]. The ainverse function and the argument selfing were used to incorporate the selfing rate (*s*) estimated from the genotyped individuals (average *s* = 0.30; see results below). The mixed model that utilised the ***A***-matrix was referred to as ABLUP-1, while the mixed model that used matrix ***A***_2_ was referred to as ABLUP-2.

In the genomic-based GBLUP approach, the average numerator relationship matrix ***A*** (***A***-matrix) derived from pedigree information in the previous mixed models (2) was substituted by the genomic relationship matrix (***G***-matrix), estimated according to VanRaden [29] and based on the 4373 SNPs:

3 $${\varvec{G}}= \frac{{\varvec{W}}{{\varvec{W}}}{\prime}}{2 \sum {p}_{i}\left(1-{p}_{i}\right)}$$

where ${\varvec{W}}$ is a matrix of order *n* x *m* (*n* = number of individuals, *m* = number of SNPs) with entries equal to ***w***_*ij*_ = ***g***_*ij*_ − 2***p***_*i*_, in which ***g***_*ij*_ is the gene content at SNP locus *i* for tree *j*, and ***p***_*i*_ is the current allele frequency for marker *i*.

Finally, in the ssGBLUP approach, the ***A***-matrix was substituted by the combined additive relationship ***H***-matrix [30; 32], resulting from combining the ***G***- with the ***A***- or ***A***_2_-matrix depending on whether inbreeding was accounted for:

4 $${\varvec{H}}=\left[\begin{array}{cc}{{\varvec{A}}}_{11 }+ {{\varvec{A}}}_{12}{{\varvec{A}}}_{22}^{-1}({{\varvec{G}}}_{{\varvec{w}}}- {{\varvec{A}}}_{22}){{\varvec{A}}}_{22}^{-1}{{\varvec{A}}}_{21}& {{\varvec{A}}}_{12}{{\varvec{A}}}_{22}^{-1}{{\varvec{G}}}_{{\varvec{w}}}\\ {{\varvec{G}}}_{{\varvec{w}}}{{\varvec{A}}}_{22}^{-1}{{\varvec{A}}}_{21}& {{\varvec{G}}}_{{\varvec{w}}}\end{array}\right]$$

where ${{\varvec{A}}}_{11}$ is the relationship matrix for non-genotyped individuals, ${{\varvec{A}}}_{22}$ is the pedigree-based relationship matrix for genotyped individuals, and ${{\varvec{A}}}_{12}$ and ${{\varvec{A}}}_{21}$ are the additive genetic relationship matrices between genotyped and non-genotyped individuals, respectively. ${{\varvec{G}}}_{{\varvec{w}}}$ is the marker-based relationship matrix for genotyped individuals weighted as: ${{\varvec{G}}}_{{\varvec{w}}}=0.90 {\varvec{G}}+0.10\boldsymbol{ }{{\varvec{A}}}_{22}$. The ***A***-matrix was combined with the ***G***-matrix to obtain the ***H***-matrix, while matrix ***A***_2_ was combined with the ***G***-matrix to obtain the ***H***_2_-matrix. The mixed model that utilised the ***H***-matrix was referred to as ssGBLUP-1, while the mixed model that used the ***H***_2_-matrix was referred to as ssGBLUP-2. The combined ***H***-matrix for the ssGBLUP analysis was obtained using the R package ASRgenomics [70].

### Genetic parameters

Restricted maximum likelihood [75] was used to estimate the (co)variances for the random effects in mixed models (1) and (2), and were obtained with the ASReml-R programme [74], which used the average information algorithm [76].

The narrow-sense individual heritability ($${h}^{2})$$ and the additive genetic correlation ($${r}_{a}$$) between the three sites were estimated as:

$${h}^{2}=\frac{{\sigma }_{a}^{2}}{{\sigma }_{a}^{2}+{\sigma }_{e}^{2}+{\sigma }_{b}^{2}};{r}_{a}= \frac{{\sigma }_{{a}_{ij}}}{\sqrt{{\sigma }_{{a}_{ii}}^{2}x {\sigma }_{{a}_{jj}}^{2}}}$$

where ${\sigma }_{a}^{2}$ is the additive genetic variance; ${\sigma }_{b}^{2}$ bulk (seed-lot) variance and ${\sigma }_{e}^{2}$ are the residual variances for each site; ${\sigma }_{{a}_{ij}}$ is the additive genetic covariance between sites *i* and *j*; and ${\sigma }_{{a}_{ii}}^{2}$ and ${\sigma }_{{a}_{jj}}^{2}$ are additive genetic variances for sites *i* and *j*, respectively, using a multi-environment model (2).

### Theoretical accuracy of breeding values and efficiency of ssGBLUP

The theoretical accuracy ($Acc$) of the predicted breeding values was estimated using the following expression:

$$Acc=\sqrt{1- \frac{PEV}{(1+ {F}_{i}){\sigma }_{a}^{2}}}$$

Where $PEV$ is the prediction error variance and is calculated as the square of the standard error; ${F}_{i}$ is the inbreeding coefficients of the *i*^th^ tree; and ${\sigma }_{a}^{2}$ is the additive genetic variance.

Efficiency ($E$) of ssGBLUP, which is the proportion of the extra benefit of GBLUP over ABLUP in the ssGBLUP scenario, was estimated following the method described in Sanchez-Mayor et al. [41].

$$E= \frac{(ssGBLUP-ABLUP)}{(GBLUP-ABLUP)}$$

When $E$ = 1, ssGBLUP has the same performance as GBLUP. However, when $E$ = 0, ssGBLUP has no advantage over ABLUP. This $E$ parameter was calculated for the theoretical accuracy of the predicted breeding values.

Finally, the expected genetic gain, i.e., the change in the average breeding value of a population after selection, was calculated as follows: 1) the net breeding value of a tree was calculated as the sum of the estimated provenance fixed effect and the predicted breeding value of the tree within the provenance [77] from Eq. (2) of the ABLUP and ssGBLUP models with and without inbreeding; 2) the average of this net breeding value of the entire population was subtracted from the average net breeding value of the top 10% of selected trees (reported as a percent increase in average breeding value).

### Predictive accuracy, prediction bias, and cross-validation scenarios

The predictive accuracy (PA) and prediction bias (PB) of all five models were evaluated using tenfold cross-validation, where 10% was used as the validation set and the remaining 90% as the training set. Two scenarios were tested to investigate the impact of provenance/population structure on PA and PB: 1) random sampling, where all measured trees were in the training population at least once; and 2) random sampling within the three provenances with strong genetic structure. The PA was determined as the Pearson correlation between the breeding values calculated from the full dataset and the ssGBLUP model (i.e., using marker and phenotype data of all the trees) and those predicted from the validation set using the ABLUP-1, ABLUP-2, ssGBLUP-1, ssGBLUP-2, and GBLUP models. PB was estimated by measuring the slope of the regression coefficient between the breeding values from the full dataset and the ssGBLUP model and those predicted with either the ABLUP-1, ABLUP-2, ssGBLUP-1, ssGBLUP-2, or GBLUP model. A regression coefficient equal to one means absence of bias.

### Supplementary Information

**Additional file 1. **

## Data Availability

Our field studies and experimental research on plants, as well as the collection of plant material, adhered to all institutional, national, and international guidelines and legislation. Voucher specimens were not obtained for the trees that were sampled and described in the manuscript. Each tree was labeled with unique identifier (from nursery to field), ensuring the preservation of their identity throughout genomic and phenotypic measurements. The phenotype, pedigree, and genotype data generated and/or analyzed during the current study are publicly available in the ERDA repository: Public Archive: 10.17894/ucph.319eafee-91a8-4405-9242-78c85f0b5d14.

## Abbreviations

ABLUP: Pedigree-based Best Linear Unbiased Prediction
ABLUP-1: ABLUP model without accounting for inbreeding
ABLUP-2: ABLUP model accounting for inbreeding
BLUE: Best Linear Unbiased Estimation
BSO: Breeding Seedling Orchard
PATSPO: Provision of Adequate Tree Seed Portfolio
DArT: Diversity Arrays Technology
GBLUP: Genomic Best Linear Unbiased Prediction
ILRI: International Livestock Research Institute
PA: Predictive Accuracy
PB: Prediction Bias
SM: Sekela Mariam
ssGBLUP: Single-step Genomic Best Linear Unbiased Prediction
ssGBLUP-1: ssGBLUP model without accounting for inbreeding
ssGBLUP-2: ssGBLUP model accounting for inbreeding
Suba: Menagesha Suba
