# Supplementary material for: Genomic evaluation for breeding and genetic management in Cordia africana, a multipurpose tropical tree species
Source: BMC Genomics. 2024 Jan 2;25:9. doi: 10.1186/s12864-023-09907-z (PMC10759591; doi:10.1186/s12864-023-09907-z)
Supplement: Supplementary file 1 — Additional file 1. [file 12864_2023_9907_MOESM1_ESM.pdf]

Table S1. Latitude (Lat., N), longitude (Long., E), altitude (Alt., masl), number of open-pollinated families phenotyped (Fam) and genotyped (GFam), and total number of individuals phenotyped (Indv.) and genotyped (GIndv.) in three provenances.

Provenances	Lat.	Long.	Alt.	Fam.	GFam.	Indiv.	GIndiv.
**North Bench (P30)**	7° 1' 48"	35° 24' 0"	1850	23	7	1276	164
**Adwa (P31)**	14° 6' 0"	38° 32' 24"	1900	20	9	2843	241
**Harar (P34)**	9° 11' 24"	42° 7' 12"	1850	20	7	1037	145
**Total**	-	-	-	**63**	**23**	**5116**	**550**

Table S2. Average predictive accuracy (PA) and prediction bias (PB) for the ABLUP, GBLUP, and ssGBLUP prediction models studied using the four combined scenarios. These scenarios included the presence (I) or absence (NI) of inbreeding and random (R) and within-provenance (P) cross-validation scenarios.

Models	Scenarios							
	I+P		I+R		NI+P		NI+R	
	PA (±SD)	PB (±SD)	PA (±SD)	PB (±SD)	PA (±SD)	PB (±SD)	PA (±SD)	PB (±SD)
**ABLUP**	0.72(0.02)	0.97(0.06)	0.66(0.03)	0.97(0.06)	0.62(0.03)	0.97(0.07)	0.56(0.03)	0.98(0.07)
**ssGBLUP**	0.73(0.02)	1.01(0.06)	0.67(0.02)	1.00(0.06)	0.63(0.02)	1.00(0.06)	0.58(0.03)	1.01(0.07)
**GBLUP**	0.58(0.07)	0.95(0.35)	0.54(0.09)	0.79(0.23)	0.58(0.08)	1.03(0.32)	0.53(0.11)	0.82(0.25)

Table S3. List of the three provenances and their families, with the total number of phenotyped trees and the number of trees sampled for genotyping.

Provenance	Family	Total number of trees	Number of trees sampled for genotyping
**North Bench**	S30	145	24
	S35	169	24
	S38	111	24
	S41	193	24
	S45	93	23
	S50	86	22
	S53	166	23
**Adwa**	S54	169	34
	S57	148	24
	S60	116	30
	S62	172	24
	S64	141	24
	S67	178	30
	S69	175	22
	S71	112	30

	S72	138	23
**Harar**	S80	125	25
	S81	102	24
	S83	91	24
	S87	56	15
	S93	59	13
	S97	188	30
	S99	34	14
**Total**		**2967**	**550**

Table S4. Summary statistics of the open-pollinated families, including the proportion of genotyped and non-genotyped individuals, along with their corresponding trait means and standard deviation.

	**Number of records**	**Mean (±SD)**	
		**Height (dm)**	**Stem at base (count)**
**Total number of offspring**	8,070	11.6 (5.4)	1.2 (0.6)
**Genotyped offspring**	490 (6.1%)	15.2 (4.3)	1.7 (0.9)
**Trees from mothers with genotyped offspring**	2958 (36.7%)	11.7 (5.4)	2.3 (0.7)
**All mothers**	63	-	-
**Mother with at least one genotyped offspring**	23	-	-

Note: dm = decimetre; SD=standard deviation; “-“= not measured

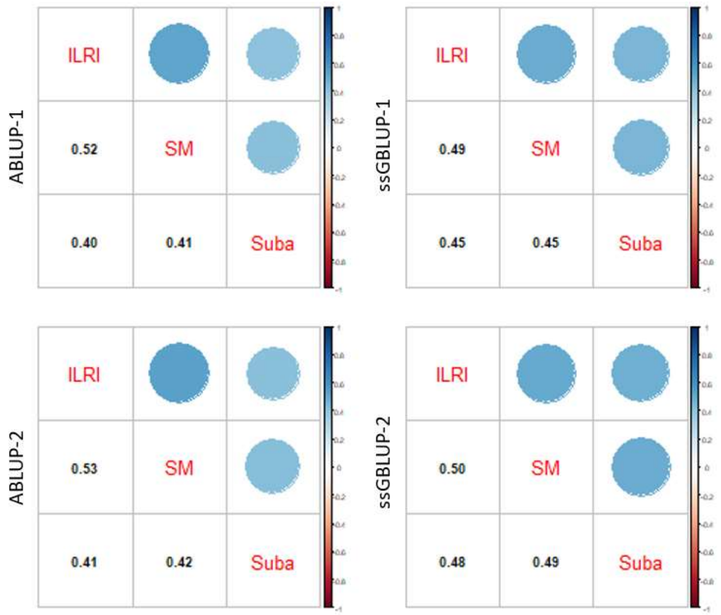

Note: ILRI = International Livestock Research Institute campus, Addis Ababa; SM = Sekela Mariam Forest; Suba = Menagesha Suba Forest.

Fig. S1: Multiple-site additive genetic correlations estimates between the three *Cordia africana* breeding seedling orchard (BSO) sites from the different models evaluated. Genetic correlation estimates are shown in each cell below the diagonal, and the light to dark blue colour of each individual cell above the diagonal reflects the strength of the genetic correlation. Abbreviations used for the models and sites are described in the text.

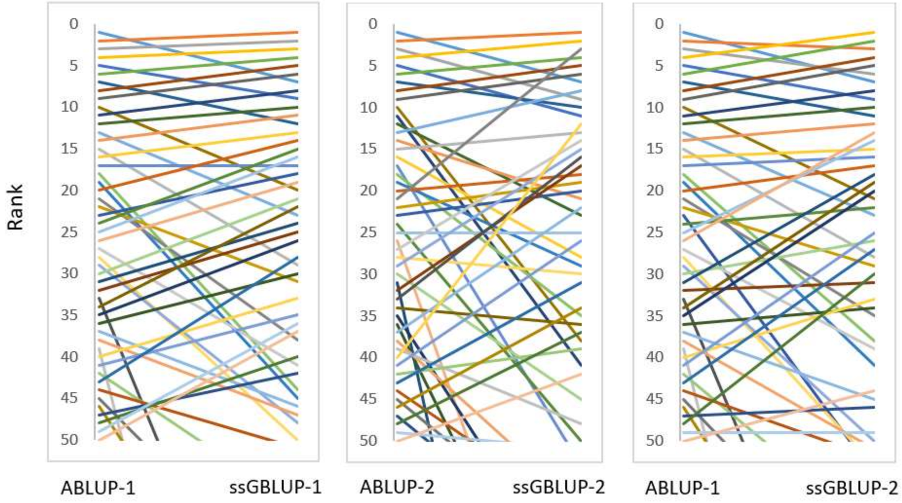

Fig. S2: Ranking plots for the top 50 *Cordia africana* genotypes comparing different ABLUP to ssGBLUP models. The compared models include those not accounting for inbreeding (left) and those that do (middle), as well as a comparison of ABLUP not accounting for inbreeding with a ssGBLUP that accounts for inbreeding (right).
